# Financial Difficulty Over Time in Young Adults With Breast Cancer

**DOI:** 10.1001/jamanetworkopen.2024.46091

**Published:** 2024-11-13

**Authors:** Sara P. Myers, Yue Zheng, Kate Dibble, Elizabeth A. Mittendorf, Tari A. King, Kathryn J. Ruddy, Jeffrey M. Peppercorn, Lidia Schapira, Virginia F. Borges, Steven E. Come, Shoshana M. Rosenberg, Ann H. Partridge

**Affiliations:** 1 Division of Breast Surgery, Department of Surgery, Brigham and Women’s Hospital, Boston, Massachusetts; 2 Breast Oncology Program, Dana-Farber Brigham Cancer Center, Boston, Massachusetts; 3 Harvard Medical School, Boston, Massachusetts; 4 Department of Medical Oncology, Dana-Farber Cancer Institute, Boston, Massachusetts; 5 Mayo Clinic, Rochester, Minnesota; 6 Massachusetts General Hospital, Boston, Massachusetts; 7 Stanford Cancer Institute, Palo Alto, California; 8 University of Colorado Comprehensive Cancer Center, Aurora; 9 Beth Israel Deaconess Medical Center, Boston, Massachusetts; 10 Division of Epidemiology, Department of Population Health Sciences, Weill Cornell Medicine, New York, New York

## Abstract

**IMPORTANCE:**

Young adults aged 18 to 39 years represent the minority of breast cancer diagnoses but are particularly vulnerable to financial hardship. Factors contributing to sustained financial hardship are unknown.

**OBJECTIVES:**

To identify financial hardship patterns over time and characterize factors associated with discrete trajectories; it was hypothesized that treatment-related arm morbidity, a key source of expense, would be associated with long-term financial difficulty.

**DESIGN, SETTING, AND PARTICIPANTS:**

This cohort study included US young adults aged 40 years or younger treated between 2006 and 2016. Eligible patients were treated for stage 0 to stage III breast cancer at institutions participating in the Young Women’s Breast Cancer Study, which included a specialized cancer institute and 12 other academic and community hospitals. Patients who responded at baseline and returned a 1-year survey were included in analysis. Data were analyzed in March 2024.

**MAIN OUTCOMES AND MEASURES:**

Trajectory modeling classified patterns of financial difficulty from baseline through 10 years postdiagnosis using the Cancer Rehabilitation Evaluation System (CARES) scale. Multinomial regression examined characteristics, including treatment-related arm morbidity, associated with each trajectory.

**RESULTS:**

A total 1008 patients were included (median [IQR] age at diagnosis, 36 [33-39] years; 60 Asian [6.0%], 35 Black [3.5%], 47 Hispanic [4.7%], 884 White [87.7%]); 840 patients were college graduates (83.3%), 764 were partnered at baseline (75.8%), 649 were nulliparous (64.4%), and 908 were without comorbidities at enrollment (90.1%). Patients’ tumors were primarily stage I-II (778 [77.2%]), estrogen receptor/progesterone receptor–positive (754 [74.8%]), and *ERBB2*-negative (formerly *HER2*) (686 [68.1%]). Patients were more frequently treated with mastectomy than breast conservation (771 [76.5%] vs 297 [29.5%]; *P* < .001). A majority of patients received radiation therapy (627 [62.2%]), chemotherapy (760 [75.4%]), and endocrine therapy (610 [60.6%]). A total of 727 patients (72.1%) reported arm symptoms within 2 years of surgery. Three distinct trajectories of experiences with finances emerged: 551 patients (54.7%) had low financial difficulty (trajectory 1), 293 (29.1%) had mild difficulty that improved (trajectory 2), and 164 (16.3%) had moderate to severe difficulty peaking several years after diagnosis before improving (trajectory 3). Hispanic ethnicity (OR, 3.71; 95% CI, 1.47-9.36), unemployment at baseline and 1 year (OR, 2.66; 95% CI, 1.63-4.33), and arm symptoms (OR, 1.77; 95% CI, 1.06-2.96) were associated with increased odds of experiencing trajectory 3. Having a college degree (OR, 0.20; 95% CI, 0.12-0.34) or being partnered (OR, 0.24; 95% CI, 0.15-0.38) were associated with increased odds of experiencing trajectory 1.

**CONCLUSION:**

In this cohort study of young adults with breast cancer, we identified a subset of patients who experienced a high degree of financial difficulty persisting into early survivorship. Targeted interventions to mitigate financial toxicity—modifiable factors that include support for the employability or return to work support for those experiencing arm symptoms after treatment—are needed.

## Key Points

**Question:**

How prevalent is financial difficulty over time following breast cancer treatment among young adults ages 40 years or younger, and what demographic and clinical characteristics are associated with financial difficulty?

**Findings:**

In this cohort study of US young adults treated for stage 0 to stage III breast cancer, 54% of patients had little to no financial difficulty, 30% had moderate financial difficulty that improved over time, and 16% had a higher degree of difficulty that persisted into early survivorship before financial recovery. High body mass index, bilateral mastectomy, Hispanic ethnicity, unemployment, and arm morbidity in follow-up were associated with trajectories involving financial difficulty.

**Meaning:**

These results suggest that targeted interventions to mitigate modifiable factors such as arm symptoms after cancer may improve long-term financial difficulty among young adults with breast cancer.

## Teaser

This cohort study examines the prevalence of and factors associated with financial hardship among US adults aged 40 years or younger treated for breast cancer.

## Introduction

Nearly 10% of new breast cancer diagnoses are made in young adults aged 18 to 39 years.^1,2,3^ Despite representing a fraction of incident cases, the proportion of advanced disease stage at presentation^4^ and aggressive tumor subtypes^5^ is high among this demographic. Existing data highlight that these disease characteristics bestow greater treatment-associated costs,^6^ indicating that the young adult population may be at elevated risk for experiencing negative consequences that result from the financial burden of their cancer care. Additionally, because young adults are often in a period of developmental growth and transition to independence, they may be contending with emotional vulnerability and fiscal instability that exacerbate economic stressors.^7^

Accordingly, young adults may be especially susceptible to financial toxicity, the multidimensional phenomenon that describes the interplay between objective material cost of cancer care and the subjective experience of cost-related psychosocial distress.^8^ Although financial toxicity after breast cancer has been well documented,^9^ our understanding as it pertains to the young adult population is incomplete. This is particularly salient for young adult patients, who may benefit most from aggressive treatment with respect to disease-free interval and overall survival. Moreover, young adults are more likely to receive multimodality treatment, which places them at risk for costly treatment-related adverse events. Specifically, young adults are more likely to be prescribed comprehensive axillary management, including both axillary lymph node dissection and regional nodal irradiation, the greatest risk factors for developing lifelong arm morbidity including breast cancer-related lymphedema. These arm-related symptoms are associated with direct and indirect costs that can contribute significantly to the economic burden of cancer care. Increased life expectancy of young adults that protracts and perpetuates the impact of treatment-related adverse events on employment and future earning potential exacerbates risk for long-term financial hardship.

In this analysis of a multi-institutional prospective cohort study of young adults, we sought to identify patterns of financial hardship over time, and characterize factors associated with sustained or increased financial burden as a first step in designing customized interventions to alleviate economic distress associated with cancer care. We were particularly interested in assessing the potential role of arm-related symptoms, with the hypothesis that arm morbidity would be associated with sustained financial difficulty.

## Methods

### Study Population

This study utilized data from Helping Ourselves, Helping Others: The Young Women’s Breast Cancer Study (YWS), a multi-institutional prospective cohort study of women aged 40 years or younger with newly diagnosed breast cancer enrolled between 2006 and 2016. Institutional review board approval was obtained at the Dana-Farber Cancer Institute and 12 other academic and community hospital sites in the US and Canada. Six of these institutions were community hospitals within Massachusetts (Cape Cod Hospital, Lowell General Hospital, Milford Hospital, Newton-Wellesley Hospital, North Shore Cancer Center [Salem], South Shore Hospital), 3 were academic medical centers in Massachusetts (Dana-Farber Cancer Institute/Brigham and Women’s Hospital, Beth Israel Deaconess Medical Center, and Massachusetts General Hospital), and 4 were academic medical centers outside of Massachusetts (Mayo Clinic in Minnesota, University of Colorado Hospital, Sunnybrook Health Sciences in Canada). Overall, the majority of patients were accrued from academic medical centers (1211 participants vs 91 from community centers). Data from the 62 Canadian participants are excluded from this analysis as they completed abbreviated surveys that did not include the items measuring the primary outcome of interest in the current study.

**Figure 1.**
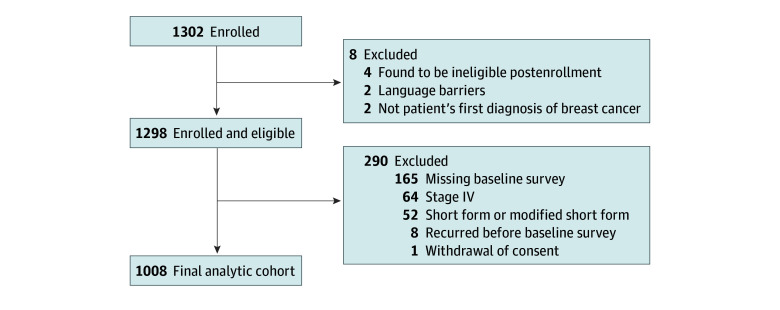
Study Design

After providing written informed consent, women enrolled in the study were asked to complete surveys at baseline, biannually for the first 3 years after diagnosis, and then annually (Figure 1). Study design details, which have previously been reported,^10^ followed the Strengthening the Reporting of Observational Studies in Epidemiology (STROBE) reporting guideline. Women with stage 0 to III breast cancer who responded to the baseline survey were eligible for inclusion in this analysis, which was performed between February 28 and March 27, 2024. Patients who experienced a breast cancer recurrence prior to their 1-year survey were excluded. Otherwise, data were censored at death, disease recurrence, new diagnosis of a second primary breast cancer, or loss to follow-up.

### Patient and Clinical Characteristics

Patient, disease, and treatment information was obtained from serially collected surveys and medical record review when indicated. Demographic variables of interest included age at diagnosis, self-reported race and Hispanic or Latino ethnicity, partner status at baseline, parity at baseline, and highest education attained at time of diagnosis. As race and ethnicity were felt to be important covariates that might impact financial toxicity,^11^ if they were unavailable or not reported on the baseline survey, they were collected from medical record review at the time of study enrollment. As research on financial toxicity has identified that financial hardship varies by age among young adults,^12,13,14^ age was categorized as follows: 18 to 25 years and 26 to 39 years. Change in employment status from baseline was assessed using a question adapted from the National Statistics Classification-Standard Occupational Classification.^15^ At baseline, participants were asked to describe their employment status at time of diagnosis as employed full time, employed part time, self-employed, unemployed for health reasons, unemployed for other reasons, and full-time homemaker. At 1 year, women were asked about their current employment and provided with these same response options. Change in employment was classified as: (1) women with any employment at baseline and 1 year, (2) women not in the workforce at baseline or 1 year, (3) women who gained employment from baseline to 1 year, or (4) women who were not employed at 1 year.

Clinical characteristics of interest included stage at diagnosis, hormone receptor positivity (defined as immunohistochemistry [IHC] levels 1% or higher)^16^ and *ERBB2* (formerly known as *HER2*) status (per American Society of Clinical Oncology [ASCO] guidelines at time of diagnosis).^17^ Treatment details including type of breast surgery (mastectomy with or without reconstruction), axillary staging procedure (sentinel lymph node biopsy and/or axillary dissection), and receipt of chemotherapy, radiation, and endocrine therapy were recorded. Comorbid conditions were self-reported at baseline; patients were classified as having a clinically significant comorbidity if they had 1 or more of the comorbid illnesses cataloged by the Charlson Comorbidity Index (CCI).^18^ Arm symptoms were assessed via 2 items on serial surveys taken from the validated Breast Cancer Prevention Trial Symptom Scale^19^; patients were asked about degree of arm swelling (lymphedema) and range of motion limitations in the ipsilateral arm after surgery in the 4 weeks preceding survey administration. Responses were recorded as a 5-point Likert scale (0, not at all; 4, extremely). Patients affirming any swelling and functional limitations on at least 1 survey from 6 months after surgery to within 2 years of diagnosis were considered to have clinically relevant arm morbidity.

### Outcomes of Interest

The primary outcome of interest, perceived financial difficulty, was assessed using a question adapted from the validated Cancer Rehabilitation Evaluation System (CARES) survey^20^: participants were asked to rate the degree to which they felt the statement “I have financial problems” applied in the 4 weeks preceding survey administration using a 5-point Likert scale (0, not at all; 4, very much). Participants who selected experiencing financial problems “a little” were considered to have mild financial difficulty. Moderate to severe financial difficulty was classified as experiencing financial problems “a fair amount,” “much,” or “very much.” This item was assessed at baseline and on serial surveys at years 1 through 5, 7, and 10.

### Statistical Analysis

Group-based trajectory modeling^21^ using a zero-inflated Poisson distribution to accommodate count data was used to classify patterns of financial difficulty from baseline through 10 years after diagnosis. The optimal number of trajectories was identified using a bayesian information criterion.^22^ Descriptive statistics were used to understand how cohort characteristics differed among patients in different trajectories. Multinomial logistic regression was used to identify the patient, disease, and treatment characteristics associated with each trajectory. Variables included in the multivariable model were selected using backward elimination. Analyses were conducted using SAS version 9.4 (SAS Institute, Inc.) with a *P* value of .05 as the threshold for significance for 2-sided hypothesis testing.

## Results

Of the 1298 eligible participants, 1008 patients were included in the final analysis (median [IQR] age at diagnosis, 36 [33-39] years; 60 Asian [6.0%], 35 Black [3.5%], 47 Hispanic [4.7%], 884 White [87.7%]) (Table 1). Response rates for surveys administered from baseline to 5 years ranged from 86% to 91%. Response rates from years 7 and 10 were lower at 71% and 65%, respectively. The majority of patients were college graduates (840 [83.3%]), partnered at baseline (764 [75.8%]), had given birth to at least 1 child (649 [64.4%]), and without clinically significant comorbidities (908 [90.1%]). Of those surveyed, 399 (39.6%) were employed at baseline and 1 year, 357 (35.4%) were not employed at either baseline or 1 year, 105 (10.4%) endorsed gaining employment between diagnosis and 1 year, and 38 (3.8%) reported losing employment; 395 of 899 patients (43.9%) who provided employment information were unemployed at 1 year.

**Table 1.** Patient and Clinical Characteristics

Characteristics	Patients, No. (%) (N = 1008)
Age at diagnosis, median (IQR), y	36 (33-39)
Age	
Emerging adults (age 17-25 y)	19 (1.9)
Young adults (age 26-40 y)	989 (98.1)
BMI	
<25	654 (64.9)
≥25	348 (34.5)
Race	
American Indian or Alaskan	4 (0.4)
Asian	60 (6.0)
Black or African American	35 (3.5)
White	884 (87.7)
>1 category reported	13 (1.3)
Other/unknown^a^	12 (1.2)
Hispanic or Latino	
Yes	47 (4.7)
No	958 (95.0)
Missing	3 (0.3)
Highest level of education at diagnosis^b^	
Non–college graduate	163 (16.2)
College graduate	840 (83.3)
Missing	5 (0.5)
Partnered at baseline	
Yes	764 (75.8)
No	237 (23.5)
Missing	7 (0.7)
Parous at diagnosis	
Yes	649 (64.4)
No	359 (35.6)
Employment status at time of diagnosis	
Employed at baseline and 1 y	399 (39.6)
Not employed at baseline or 1 y	357 (35.4)
Gained employment at 1 y	105 (10.4)
Lost employment at 1 y	38 (3.8)
Unknown or censored	109 (10.8)
Any comorbid condition	100 (9.9)
Stage at diagnosis	
0	86 (8.5)
I	354 (35.1)
II	424 (42.1)
III	144 (14.3)
ER/PR positive	
Yes	754 (74.8)
No	253 (25.1)
Missing	1 (0.1)
*ERBB2* positive	
Yes	273 (27.1)
No	686 (68.1)
Missing	49 (4.9)
Surgery	
Lumpectomy	297 (29.5)
Unilateral mastectomy	255 (25.3)
Bilateral mastectomy	456 (45.2)
Radiation	
Yes	627 (62.2)
No	381 (37.8)
Chemotherapy	
Yes	760 (75.4)
No	248 (24.6)
Endocrine therapy^c^	
Yes	630 (62.5)
No	376 (37.3)
Missing	2 (0.2)
Arm morbidity within 2 y of diagnosis^d^	
Yes	727 (72.1)
No	280 (27.8)
Missing	1 (0.1)

Abbreviations: BMI, body mass index (calculated as weight in kilograms divided by height in meters squared); ER, estrogen receptor; PR, progesterone receptor.

^a^ Other race includes American Indian, Alaskan Native, Native Hawaiian, and Pacific Islander.

^b^ Non–college degree includes some college and those with vocational training; college graduate includes those with postcollege graduate degrees or professional degrees.

^c^ Any endocrine therapy from baseline to 1 year.

^d^ Arm morbidity as defined by patient-reported composite of arm swelling and decreased range of motion.

Patients’ tumors were predominantly stage 1 or 2 (354 stage I [35.1%], 424 stage II [42.1%]), estrogen receptor/progesterone receptor (ER/PR)-positive (754 [74.8%]), and *ERBB2*-negative (686 [68.1%]). Patients were more frequently treated with mastectomy (unilateral or bilateral, 711 [70.5%]) than breast conservation. Receipt of radiation (627 [62.2%]), chemotherapy (760 [75.4%]), and endocrine therapy (630 [62.5%]) was common. A total of 721 patients (71.5%) complained of either arm swelling or limited range of motion on the side of their surgery within 2 years of their definitive cancer operation.

**Figure 2.**
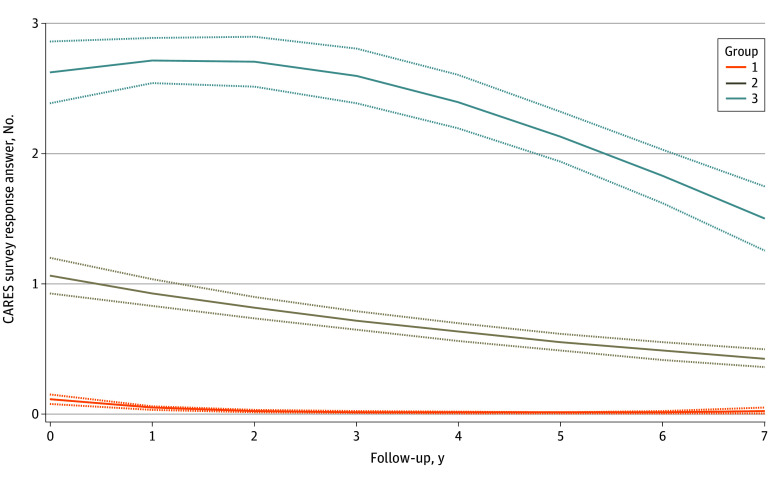
Trajectories of Financial Difficulty Over Time Compared with 54% of patients that experienced little or no financial difficulty (group 1), breast cancer-related lymphedema was associated with moderate (group 2) or severe (group 3) financial difficulty that persisted into survivorship in 30% and 17% of patients, respectively.

Overall, 80% of women reported no financial difficulty at 10 years, and for the majority of patients the prevalence of perceived financial difficulty diminished as time from cancer diagnosis elapsed (eFigure in Supplement 1). Group-based trajectory modeling identified that the data were best fit by a 3-trajectory solution describing trends in financial difficulty over time (Figure 2). The majority of patients (551 [54.7%]) had a stable and low degree of financial difficulty (ie, no financial difficulty, trajectory 1); nearly a third of patients (293 [29.1%]) had a mild degree of financial difficulty that gradually improved (trajectory 2); and 164 (16.3%) experienced a moderate to severe degree of financial difficulty at baseline, which peaked several years after diagnosis followed by a decline toward less financial difficulty (trajectory 3).

Age, race, parity at baseline, comorbidity, clinical stage, tumor subtype, receipt of radiation, and systemic therapy were not significantly associated with trajectory affiliation, and were therefore eliminated from the final model (Table 2). Multinomial regression identified several independent associations between clinical and disease characteristics and financial trajectories. Elevated body mass index (BMI; calculated as weight in kilograms divided by height in meters squared) of 25 or higher (trajectory 3 vs trajectory 1: OR, 2.59; 95% CI, 1.68-4.00), bilateral mastectomy (OR, 2.00; 95% CI, 1.19-3.36), Hispanic ethnicity (OR, 3.71; 95% CI, 1.47-9.36), being unemployed at baseline and at 1 year (OR, 2.66; 95% CI, 1.63-4.33), and having arm symptoms (OR, 1.77; 95% CI, 1.06-2.96) were associated with financial difficulty trajectories (Table 3). Having a college degree or being partnered were protective against membership in both financial difficulty trajectories.

**Table 2.** Patient and Clinical Characteristics by Trajectory

Characteristics	Patients, No. (%)		
	Trajectory 1 (N = 551)	Trajectory 2 (N = 293)	Trajectory 3 (N = 164)
Age at diagnosis, median (IQR), y	37 (33-39)	36 (33-39)	37 (32.5-39)
Age			
Emerging adults (age 17-25)	6 (1.1)	9 (3.1)	4 (2.4)
Young adults (age 26-40)	545 (98.9)	284 (96.9)	160 (97.6)
BMI			
<25	395 (71.7)	182 (62.1)	77 (47.0)
≥25	154 (28.0)	109 (37.2)	85 (51.8)
Race			
American Indian or Alaskan	1 (0.2)	2 (0.7)	1 (0.6)
Asian	38 (6.9)	12 (4.1)	10 (6.1)
Black of African American	12 (2.2)	14 (4.8)	9 (5.5)
White	490 (88.9)	263 (89.8)	131 (79.9)
>1 category reported	4 (0.7)	2 (0.7)	7 (4.3)
Other or unknown^a^	6 (1.1)	0	6 (3.7)
Hispanic or Latino			
Yes	15 (2.7)	18 (6.1)	14 (8.5)
No	536 (9.2)	274 (93.5)	148 (90.2)
Missing	0	1 (0.3)	2 (1.2)
Highest level of education at diagnosis^b^			
Non–college graduate	49 (8.9)	56 (19.1)	58 (35.4)
College graduate	502 (91.1)	235 (80.2)	103 (62.8)
Missing	0	2 (0.7)	3 (1.8)
Partnered at baseline			
Yes	456 (82.8)	216 (73.7)	92 (56.1)
No	92 (16.7)	76 (25.9)	69 (42.1)
Missing	3 (0.5)	1 (0.3)	3 (1.8)
Parous at diagnosis			
Yes	349 (63.3)	199 (67.9)	101 (61.6)
No	202 (36.7)	94 (32.1)	63 (38.4)
Employment status at time of diagnosis			
Employed at baseline and 1 y	246 (44.7)	110 (37.5)	43 (26.2)
Not employed at baseline or 1 y	186 (33.8)	110 (37.5)	61 (37.2)
Gained employment at 1 y	58 (10.5)	27 (9.2)	20 (12.2)
Lost employment at 1 y	16 (2.9)	12 (4.1)	10 (6.1)
Unknown or censored	45 (8.2)	34 (11.6)	30 (18.3)
Any comorbid condition	46 (8.4)	30 (10.2)	24 (14.6)
Stage at diagnosis			
0	52 (9.4)	22 (7.5)	12 (7.3)
I	210 (38.1)	98 (33.5)	46 (28.1)
II	213 (38.7)	133 (45.4)	78 (47.6)
III	76 (13.8)	40 (13.7)	28 (17.1)
ER/PR positive			
Yes	414 (75.1)	218 (74.4)	122 (74.4)
No	137 (24.9)	74 (25.3)	42 (25.6)
Missing	0	1 (0.3)	0
*ERBB2* positive			
Yes	157 (28.5)	72 (24.6)	44 (26.8)
No	366 (66.4)	207 (70.7)	113 (68.9)
Missing	28 (5.1)	14 (4.8)	7 (4.3)
Surgery			
Lumpectomy	168 (30.5)	85 (29.0)	44 (26.8)
Unilateral mastectomy	147 (26.7)	72 (24.6)	36 (22.0)
Bilateral mastectomy	236 (42.8)	136 (46.4)	84 (51.2)
Radiation			
Yes	339 (61.5)	188 (64.2)	100 (61.0)
No	212 (38.5)	105 (35.8)	64 (39.0)
Chemotherapy			
Yes	397 (72.1)	223 (76.1)	140 (85.4)
No	154 (28.0)	70 (23.9)	24 (14.6)
Endocrine therapy^c^			
Yes	352 (63.9)	164 (62.8)	94 (57.3)
No	197 (35.8)	109 (37.2)	70 (42.7)
Missing	2 (0.4)	0	0
Arm morbidity within 2 y of diagnosis^d^			
Yes	375 (68.1)	216 (73.7)	130 (79.3)
No	172 (31.2)	76 (25.9)	31 (18.9)
Missing	4 (0.7)	1 (0.3)	3 (1.8)

Abbreviations: BMI, body mass index (calculated as weight in kilograms divided by height in meters squared); ER, estrogen receptor; PR, progesterone receptor.

^a^ Other race includes American Indian, Alaskan Native, Native Hawaiian, and Pacific Islander.

^b^ Non–college degree includes some college and those with vocational training; college graduate includes those with postcollege graduate degrees or professional degrees.

^c^ Any endocrine therapy from baseline to 1 year.

^d^ Arm morbidity as defined by patient-reported composite of arm swelling and decreased range of motion.

**Table 3.** Multivariate Multinomial Model of Factors Associated With Trajectory Membership

Characteristic	Trajectory comparison	OR (95% CI)	*P* value
BMI ≥25 vs <25	2 vs 1	1.43 (1.02-2.01)	.04
	3 vs 1	2.59 (1.68-4.00)	<.001
Hispanic ethnicity	2 vs 1	2.51 (1.13-5.61)	.02
	3 vs 1	3.71 (1.47-9.36)	.006
College degree	2 vs 1	0.40 (0.25-0.63)	<.001
	3 vs 1	0.20 (0.12-0.34)	<.001
Partnered at baseline	2 vs 1	0.50 (0.34-0.73)	<.001
	3 vs 1	0.24 (0.15-0.38)	<.001
Employment status			
Unemployed at baseline and 1 y	2 vs 1	1.49 (1.05-2.11)	.02
	3 vs 1	2.66 (1.63-4.33)	<.001
Gained employment at 1 y	2 vs 1	1.00 (0.59-1.71)	>.99
	3 vs 1	1.61 (0.80-3.21)	.18
Lost employment at 1 y	2 vs 1	1.60 (0.71-3.62)	.26
	3 vs 1	3.07 (1.14-8.26)	.03
Surgery			
Unilateral mastectomy vs lumpectomy	2 vs 1	0.89 (0.58-1.38)	.61
	3 vs 1	1.04 (0.56-1.95)	.90
Bilateral mastectomy vs lumpectomy	2 vs 1	1.17 (0.81-1.70)	.40
	3 vs 1	2.00 (1.19-3.36)	.009
Arm morbidity	2 vs 1	1.36 (0.96-1.94)	.09
	3 vs 1	1.77 (1.06-2.96)	.03

Abbreviations: OR, odds ratio; BMI, body mass index (calculated as weight in kilograms divided by height in meters squared).

## Discussion

In this longitudinal survey study of young adults with breast cancer, patients’ experiences with financial difficulty followed 1 of 3 trajectories. Most patients either had little to no financial difficulty, approximately a third had a moderate degree of financial difficulty that improved gradually over time, and a minority (16.3%) had a higher degree of difficulty that persisted into early survivorship before some financial recovery. BMI 25 or higher, bilateral mastectomy, Hispanic ethnicity, being unemployed both at baseline and 1 year, and arm morbidity in follow-up were all identified as factors independently associated with trajectories involving financial difficulty.

This study corroborates others’ work describing the treatment-related economic hardship. With spending of more than $30 billion annually, breast cancer care represents a significant burden to the US economy.^23^ Increases in cost-sharing have resulted in patients assuming significant financial hardship while simultaneously navigating their breast cancer treatment. As part of a longitudinal cohort study of 1502 women, Jagsi et al^24^ reported that a third of participants experienced financial decline at 4 years of follow-up. Similar findings were observed by Greenup et al^25^ in their survey-based study of participants recruited from the Dr Susan Love Research Foundation’s Army of Women and Sisters Network of North Carolina; 35% of women reported experiencing financial burden as a result of their cancer care at a median of 6.7 years from diagnosis. Our analysis of financial difficulty over time expands upon these investigations and existing research^26,27^ by highlighting how the burden associated with the cost of breast cancer care can evolve over time. Unlike the aforementioned studies that highlight persistent burden using a cross-sectional approach, our study shows a gradual improvement of financial difficulty over time. Nearly half of the patients included in trajectory analysis experienced financial difficulty. While it is reassuring that for the majority of these individuals improvement began soon after treatment, the consequences of expensive cancer care endured for more than a third of participants. The lasting effects of financial hardship warrant consideration as resources for financial assistance and strategies to offset out-of-pocket spending may be limited after the active stage of treatment.

Several factors were found to be associated with delayed recovery from economic hardship. Others have reported that locoregional and systemic treatment strategies influence out-of-pocket spending and medical debt.^22,24,28^ While undergoing bilateral mastectomy compared with lumpectomy was associated with sustained financial difficulty, tumor and systemic treatment were not among the factors found to impact likelihood of belonging to one trajectory over another in this study. We postulate that restricting the study cohort to young adults who are more likely to receive aggressive and comprehensive treatment may have contributed to homogeneity in clinical factors that have been shown to be associated with financial hardship when a wider range of ages is considered. Of the factors that were found to be significantly associated with trajectories indicative of financial difficulty, the importance of arm morbidity highlights that treatment-related adverse events might serve as one focus of interventions for mitigating financial toxicity. In this study, arm morbidity was used as a surrogate for breast cancer–related lymphedema (BCRL), a lifelong ailment experienced by more than 20% of patients.^29^ Costs directly related to BCRL include diagnostic testing, treatments, and hospitalizations from cascading sequelae (eg, cellulitis). Out-of-pocket spending is estimated to be 7 times higher for patients who experience BCRL compared with those who do not develop this complication.^30^ The true economic impact of BCRL is likely much greater given indirect costs from associated disability, unemployment, and foregone opportunities.^31^ These issues are particularly relevant for young adults, as vocational disruption can thwart earning potential and perpetuate existing financial hardship. Although there are inconsistencies between previous studies with regard to the whether risk varies by age,^32^ young adults are more likely to receive aggressive multimodality treatment with comprehensive axillary management (ie, axillary lymph node dissection and regional nodal irradiation), which is the greatest risk factor for the development of BCRL.^33^ Additionally, our group has demonstrated previously in a cross-sectional single institution study that young age and arm morbidity are independently associated with long-term financial difficulty.^34^ The findings of this longitudinal cohort investigation strengthen our understanding of the influence of treatment-related adverse events on financial well-being and suggest that reducing the prevalence and effects of BCRL may mitigate long-term financial toxicity.

### Limitations

This study has several limitations. The homogeneity of the YWS cohort with respect to race, ethnicity, and level of educational attainment restrict generalizability of our findings. In addition, although the present study did not measure financial toxicity or specify that financial hardship was directly attributable to cancer care, others have used the CARES survey to understand the financial impact of cancer care.^35^ We also did not assess all premorbid treatment-related adverse events, which may have influenced short- and long-term comorbidity and financial status. While parity at time of diagnosis was assessed, surveys did not inquire about number of dependents or nonbiological children, both of which may contribute to financial hardship. Although data on postmastectomy reconstruction was collected, because timing and associated complications that may have occurred were incompletely evaluated, it was not included as a factor in our analysis. While investigations to date indicate that financial hardship may be impacted more by decision for mastectomy relative to breast conservation and less by whether reconstruction is pursued,^24^ we acknowledge that complications or extended recovery may impact return to work and financial difficulty. Finally, while the association between arm morbidity and financial difficulty was a key finding in this study, swelling and functional limitations were self-reported and may have overestimated the degree of patients with clinically significant arm symptoms or confirmed BCRL.

## Conclusions

In this cohort study of young adults with breast cancer, we identified a subset of patients who experienced a high degree of financial difficulty that persisted into early survivorship. Our findings contribute significantly to understanding the experiences of young breast cancer survivors and have important implications for future interventions aimed at improving patient-centered outcomes.

## Notes

Supplement 1. **eFigure.** Prevalence of Financial Difficulty as Indicated by 5-Point Likert Scale Response Indicating the Degree to Which Participant Experiences Financial Problems

Supplement 2.
Data Sharing Statement
