# Supplementary material for: Financial Difficulty Over Time in Young Adults With Breast Cancer
Source: JAMA Netw Open. 2024 Nov 13;7(11):e2446091. doi: 10.1001/jamanetworkopen.2024.46091 (PMC11561695; doi:10.1001/jamanetworkopen.2024.46091)
Supplement: Supplement 1. — eFigure. Prevalence of Financial Difficulty as Indicated by 5-Point Likert Scale Response Indicating the Degree to Which Participant Experiences Financial Problems [file jamanetwopen-e2446091-s001.pdf]

Supplemental Online Content

Myers SP, Zheng Y, Dibble K, et al. Financial difficulty after breast cancer. *JAMA Netw Open.* 2024;7(11):e2446091. doi:10.1001/jamanetworkopen.2024.46091

eFigure. Prevalence of Financial Difficulty as Indicated by 5-Point Likert Scale Response Indicating the Degree to Which Participant Experiences Financial Problems

This supplemental material has been provided by the authors to give readers additional information about their work.

© 2024 Myers SP et al. *JAMA Network Open.*

eFigure. **Prevalence of financial difficulty as indicated by 5-point Likert scale response indicating the degree to which participant experiences financial problems.**

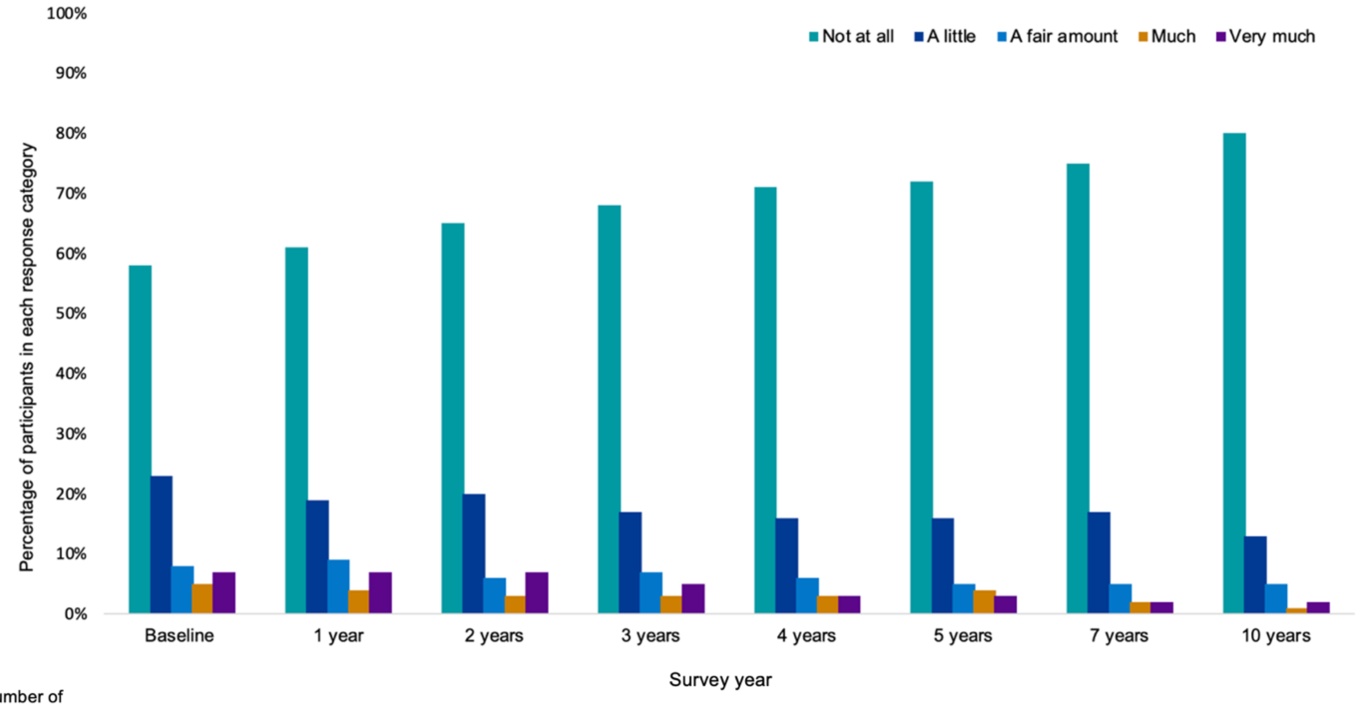

Number of participants	Baseline	1 year	2 years	3 years	4 years	5 years	7 years	10 years
Responded	998	925	824	742	695	663	629	512
Did not respond	10	83	184	266	313	245	379	496

© 2024 Myers SP et al. *JAMA Network Open.*
