# Supplementary material for: Financial Difficulty Over Time in Young Adults With Breast Cancer
Source: JAMA Netw Open. 2024 Nov 13;7(11):e2446091. doi: 10.1001/jamanetworkopen.2024.46091 (PMC11561695; doi:10.1001/jamanetworkopen.2024.46091)
Supplement: Supplement 2. — Data Sharing Statement [file jamanetwopen-e2446091-s002.pdf]

Data Sharing Statement

Myers. Financial Difficulty Over Time in Young Adults With Breast Cancer. *JAMA Netw Open*. Published November 13, 2024. doi:10.1001/jamanetworkopen.2024.46091

Data
Data available: Yes
Data types: Deidentified participant data
How to access data: The data that support these findings are available on written request to and approval from the senior author, Ann H. Partridge.
When available: With publication

Supporting Documents
Document types: None

Additional Information
Who can access the data: Researchers whose proposed use of the data has been approved
Types of analyses: For academic purposes
Mechanisms of data availability: After approval of a proposal
